# Strong correlations between power-law growth of COVID-19 in four continents and the inefficiency of soft quarantine strategies

**DOI:** 10.1063/5.0009454

**Published:** 2020-04-28

**Authors:** Cesar Manchein, Eduardo L. Brugnago, Rafael M. da Silva, Carlos F. O. Mendes, Marcus W. Beims

**Affiliations:** 1 Departamento de Física, Universidade do Estado de Santa Catarina, 89219-710 Joinville, SC, Brazill; 2 Departamento de Física, Universidade Federal do Paraná, 81531-980 Curitiba, PR, Brazil; 3 Escola Normal Superior, Universidade do Estado do Amazonas, 69050-010 Manaus, AM, Brazil

## Abstract

In this work, we analyze the growth of the cumulative number of confirmed infected cases by a novel coronavirus (COVID-19) until March 27, 2020, from countries of Asia, Europe, North America, and South America. Our results show that (i) power-law growth is observed in all countries; (ii) by using the distance correlation, the power-law curves between countries are statistically highly correlated, suggesting the universality of such curves around the world; and (iii) *soft* quarantine strategies are inefficient to flatten the growth curves. Furthermore, we present a model and strategies that allow the government to reach the flattening of the power-law curves. We found that besides the social distancing of individuals, of well known relevance, the strategy of *identifying* and *isolating* infected individuals in a large daily rate can help to flatten the power-laws. These are the essential strategies followed in the Republic of Korea. The high correlation between the power-law curves of different countries strongly indicates that the government containment measures can be applied with success around the whole world. These measures are scathing and to be applied as soon as possible.

Since the identification of a novel coronavirus (COVID-19) in Wuhan, China, in December 2019, the virus kept spreading around the world. One of the most remarkable characteristics of COVID-19 is its high infectivity, resulting in a global pandemic. In this complex scenario, tasks such as protecting the people from the infection and the global economy are considered two major challenges. In order to improve our knowledge about COVID-19 and its behavior in different countries around the world, we exhaustively explore the real time-series of cumulative number of confirmed infected cases by COVID-19 in the last few months until March 27, 2020. In our analysis, we considered the Asia, Europe, North America, and South America. Our main findings clearly show the existence of a well established power-law growth and a strong correlation between power-law curves obtained for different countries. These two observations strongly suggest a universal behavior of such curves around the world. To improve our analysis, we use a model with six autonomous ordinary differential equations, based on the well-known SEIR (Susceptible–Exposed–Infectious–Recovered) epidemic model (considering quarantine procedures), to propose efficient strategies that allow the government to increase the flattening of the power-law curves. Additionally, we also show that soft measures of quarantine are inefficient to flatten the growth curves.

## I. INTRODUCTION

The astonishing increase of positive diagnosed cases due to COVID-19 has caught the attention of the whole world, including researchers of many areas and governments. It is urgent^1^ to find explanations for the already known data and models that may allow us to better understand the evolution of the virus. Such explanations and models can hopefully be used to implement social policies and procedures to decrease the number of infections and deaths. Time is essential to avoid economic and social catastrophes.

In general, the average reproductive number $R_{0}$, which gives the number of secondary infected individuals generated by a primary infected individual, is the key quantity which determines the dynamical evolution of the epidemic.^2^ Usually, for values $R_{0}<1$, the number of new infected individuals decreases exponentially. For $1<R_{0}<\infty$, this number increases exponentially.^2,3^ However, the nature is full of surprises, and there are plenty of cases for which the exponential behavior is substituted by power-law^4^ and are related to branching processes with diverging reproductive number,^2^ scale free networks, and small worlds.^5^ It was already suggested in the literature that the COVID-19 growth might be a small world.^6^ This is in agreement with recent results^7^ suggesting that, for many countries around the world, the COVID-19 growth has the tendency to follow the power-law.

In fact, recent analysis regarding the behavior of the COVID-19 in China demonstrated a power-law $t^{\mu }$ growth of infected cases.^8^ The authors found exponents around μ=2.1±0.3, which do not vary very much for different provinces in China. This suggests that socioeconomic differences, local geography, differences in containment strategies, and heterogeneities essentially affect the *value* of the exponent μ but not the qualitative behavior. A model of coupled differential equations, which includes quarantine and isolation effects, was used by the authors to match real data. Power-law growths for China were also obtained in another study and a possible relation to fractal kinetics and graph theory is discussed.^9^

In line with the above publications, the present work analyzes the time-series evolution of the COVID-19 for the following countries: Brazil, China, France, Germany, Italy, Japan, Republic of Korea, Spain, and the United States of America (USA). In all cases, we observe a power-law increase for the positive detected individuals, where the exponent μ changes for different countries. In addition to the power-law behavior, we also computed the Distance Correlation (DC)^10^ between pairs of countries. The DC is able to detect nonlinear correlations between data.^11,12^ We show that power-law data are highly correlated between all analyzed countries. This strongly suggest that government strategies to flatten the power-law growth, valid for one country, can be successfully applied to other countries and continents. Furthermore, a model of Ordinary Differential Equations (ODEs) is proposed and some strategies to flatten the power-law curves are discussed using the numerical simulations.

The paper is divided as follows. Section II presents the power-law growth of confirmed infected cases of COVID-19 and the DC between pairs of countries is determined. Section III discusses numerical results using the proposed model showing many strategies to flatten the power-law growth. In Sec. IV, we summarize our results.

## II. REAL DATA ANALYSIS

### A. Power-law growths

**FIG. 1.**
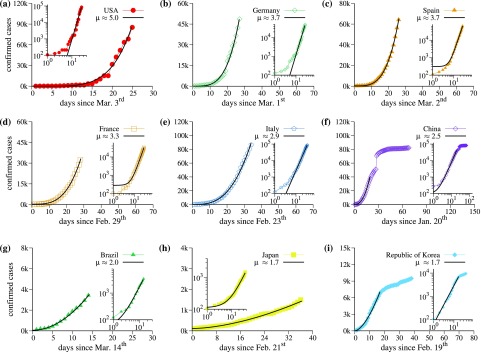
Cumulative number of confirmed infected cases by COVID-19 as a function of time for (a) USA, (b) Germany, (c) Spain, (d) France, (e) Italy, (f) China, (g) Brazil, (h) Japan, and (i) Republic of Korea, excluding days with <100 infected cases. The black-continuous curves represent the function $\alpha +\beta t^{\mu }$ that fit the time-series, and the parameters α, β, and μ for each country are described in Table I.

Figure 1 displays data of the cumulative number of confirmed positive infected cases by COVID-19 of nine countries as a function of the days. The analyzed countries are (in alphabetic order): Brazil, China, France, Germany, Italy, Japan, Republic of Korea, Spain, and the USA. Data were collected from the situation reports published daily by the World Health Organization (WHO).^13^ We notice that the values in the vertical axis in Fig. 1 change for different countries. Initial data regarding the incubation time were discarded since they do not contribute to the essential results discussed here. Black-continuous curves are the corresponding fitting curves $\alpha +\beta \;t^{\;\mu }$, where t is the time given in days and α, β, and μ are parameters. Insets in all plots show the data in the log–log scale. Straight lines in the log–log plot represent the power-law growth. The fact that the growth increases as a power-law is good news since it increases slower than the exponential one. However, that is not good enough.

**TABLE I.** Details about the parameters of the fitting curves for the power-law behavior *α* + *β* *t*^*μ*^ shown in Fig. 1.

Country	*α*	*β*	*μ*
USA	0	0.009	4.994 ± 0.216
Germany	0	0.223	3.734 ± 0.107
Spain	308	0.386	3.686 ± 0.037
France	280	0.467	3.341 ± 0.031
Italy	0	2.868	2.934 ± 0.040
China	98	24.013	2.492 ± 0.020
Brazil	59	18.450	1.971 ± 0.054
Japan	112	3.107	1.685 ± 0.034
Republic of Korea	0	62.574	1.670 ± 0.065

The regimes with power-law growth are the most relevant to be discussed since they provide essential information of what is expected for the future and possible attitudes needed to flatten the curves. The exponent μ changes for distinct countries and the complete fitting parameters are given in Table I. Results in Table I are presented in decreasing order of the exponent μ; the USA [Fig. 1(a)] has by far the largest exponent and, therefore, became already the country with epidemic records. Even though Germany [Fig. 1(b)] reported a small number of deaths, it has the second large exponent, followed by Spain [Fig. 1(c)], France [Fig. 1(d)], and Italy [Fig. 1(e)], in this order. China [Fig. 1(f)], Brazil [Fig. 1(g)], Japan [Fig. 1(h)], and Republic of Korea [Fig. 1(i)], in this order, are the last in the list. In the case of China and Republic of Korea, the power-laws are more clear due to the number of available data. For these two countries, a flatten is observed after the power-law. The jump observed after 30 days in China data is due to a change in the counting procedure of infected cases (see the situation report on the February 17, 2020, in Ref. 13). Republic of Korea, on the other hand, focused on identifying infected patients immediately and isolating them to interrupt transmission.^14^ It is interesting to note that for Japan, another country that adopted similar measures, we obtained a similar value for μ.

The most desired behavior is that if the exponent μ becomes smaller it leads to the flattening of the curves. But, this is apparently not that easy. Besides USA and Germany, which have a distinct inclination in the beginning of their power-laws, and China and Republic of Korea, which are stabilizing the epidemic spread, for all other countries the growth remains strictly on the fitted curve and μ essentially does not change in time. In Sec. III, we discuss some possibilities to flatten the power-laws.

### B. Distance correlation between countries

The power-law observed in all cases from Fig. 1 is certainly not a coincidence but a consequence of virus propagation in scale free systems. To quantify the relation between the power-law growth, we use the DC, which is a statistical measure of dependence between random vectors.^^10,15–18^^ Please do not confuse the word distance with the geographical distance between the analyzed countries. The most relevant characteristics of DC is that it will be zero if and only if the data are independent and equal to one for maximal correlation between data. Details about the numerical procedure to obtain the DC are given in Appendix A.

**FIG. 2.**
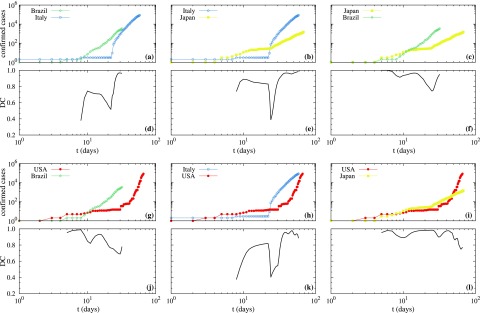
In panels (a), (b), (c), (g), (h), and (i) the log–log plot of the cumulative numbers of confirmed infected cases as a function of time is presented for the possible pairs of countries formed between Brazil, Italy, Japan, and USA. The semi-log plot of DC calculated between these pairs of countries is presented in panels (d), (e), (f), (j), (k), and (l), respectively.

Figure 2 presents specific results for the DC calculated between some selected countries, namely, Brazil, Italy, Japan, and USA. Italy was chosen due to their relevance to Europe, regarding the typical data of the virus. USA was chosen for being the top affected country, and Brazil and Japan represent distinct continents and distinct epidemic containment measures. Thus, we compute the DC between four continents. Figures 2(a)–2(c), 2(g), 2(h), and 2(i) are the cumulative number of confirmed cases in each country, as in Fig. 1, but considering data since the first day the infection was reported. In these curves, we clearly see the initial plateaus due to the incubation time. After the plateaus, a qualitative change to the power-law growth (the same from Fig. 1) occurs. The time for which the qualitative change occurs is distinct for each country.

Figures 2(d)–2(f), 2(j), 2(k), and 2(l) display the corresponding DC calculated between the countries. Results show that DC between the curves is relatively high in the beginning. The lowest values for the DC are obtained between Brazil and Italy, in Fig. 2(d), and between Italy and USA, shown in Fig. 2(k); in both cases, DC is around 0.4. DC decays substantially when the power-law starts in one country but not in the other. The exception is between Japan and USA. After some days, when both countries reach the power-law behavior, the values of DC become very close to 1. Thus, they are *highly* correlated besides distinct exponents μ. Furthermore, the DC is not necessarily related to the exponent μ. One example can be mentioned. Even though USA has the largest exponent and Japan the lowest one (considering the error in Table I), they are highly correlated. Besides that a even though there are not many data available for Brazil, it seems to become more and more correlated with Italy and Japan.

## III. PREDICTIONS AND STRATEGIES

The model proposed in this work for the numerical prediction and strategies is presented in detail in Appendix B. It is a variation of the well known Susceptible–Exposed–Infectious–Recovered (SEIR) epidemic model^19,20^ to propose efficient strategies that allow the government to increase the flattening of the power-law curves. Our SEIR model takes into account the isolation of infected individuals.^^8,21–23^^ In this case, quarantine means the identification and isolation of infected individuals. The parameters are divided in two categories: (i) those related to the characteristic of the virus spreading, defined *a priori* from other studies and (ii) those related to adjusting the model to the real data (for more details, see Appendix B). These parameters can change according to social actions and government strategies.

Numerical results of this section take into account possible interferences or strategies from the government of each country, what means that some parameters must be changed after the last day of the real data. For each distinct strategy, we use distinct colors, which are then plotted.

**FIG. 3.**
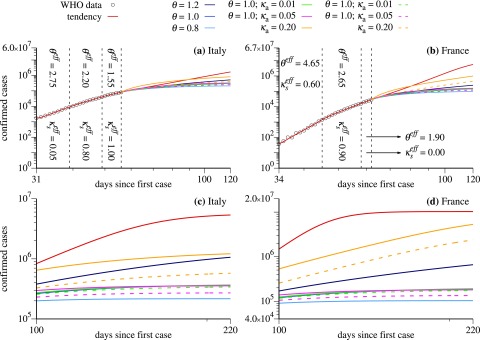
Log–log plot of cumulative number of confirmed cases (black circles) for Italy [(a) and (c)] and France [(b) and (d)] as a function of time and projected number of cases (colored lines) using distinct government strategies (discussed in the text).

For a detailed explanation of variables and parameters, see Appendix B. The colors used in Fig. 3 for the distinct scenarios are the following (for continuous curves):

*Red curves*: the tendency which follows from the behavior of the last points of real data (last values for $\theta^{eff}$ and $\kappa_{s}^{eff}$). This is what happens if we do not change the current scenario on March 28th.

*Blue curves*: reduction of social interactions by using smaller values of θ. Dark blue for θ=1.2, medium blue for θ=1.0, and light blue for θ=0.8.

*Green curves*: reduction of social interaction together with tests to identify and isolate asymptomatic and mild symptomatic cases. Here we use θ=1.0 and $\kappa_{a}=0.01$.

*Magenta curves*: reduction of social interaction together with tests to identify and isolate asymptomatic and mild symptomatic cases. Here, we use θ=1.0 and $\kappa_{a}=0.05$.

*Orange curves*: identification and isolation of asymptomatic and mild symptomatic cases with rate $\kappa_{a}=0.20$. In this strategy, we do not increase the social distance and use the last value for θ obtained in the adjustment.

For the dashed curves, the configurations are the same inside each color. However, in these curves, the asymptomatic and mild symptomatic identified cases are not accounted for. We notice that without the realization of tests in the population, the asymptomatic individuals would not be computed.

We start discussing the cases of Italy and France, shown in Figs. 3(a)–3(d), respectively. In these cases, $\kappa_{s}=1$, which means that we assume that all symptomatic individuals are properly isolated. Figures 3(c) and 3(d) show the evolution of scenarios for 120 days. The vertical axis is the cumulative number of positive infected individuals in the population. In the horizontal axis, we have the days since the first computed case in these countries. Black circles are the real data starting from the power-law-like behavior discussed in Sec. II. During the times for which real data are available, the model chooses the values of the parameters θ and $\kappa_{s}$ that better adjust the simulation results with the data. In the cases shown in Fig. 3, we needed three values of θ and $\kappa_{s}$, namely, the values $\theta^{eff}$ and $\kappa_{s}^{eff}$ given in the figures. As a consequence, the red curves are in full agreement with the data in this time interval. When the available data end, the simulation continues and the red curves can be used to predict the asymptotic number of confirmed cases since they represent the scenario following the tendency demonstrated by the data. In the case of Italy, we obtain $5.6\times 10^{6}$ and for France, $1.0\times 10^{7}$. See the tendencies in Figs. 3(c) and 3(d). The considerable difference between these projections is explained by the last values of $\theta^{eff}$ and $\kappa_{s}^{eff}$ obtained for these countries. Besides $\theta^{eff}$ being larger for France, we obtain $\kappa_{s}^{eff}=0$, which can be interpreted as the nonexistence of quarantine measures or the inefficient isolation of symptomatic individuals. We are aware that such asymptotic behavior can be hardly trusted with numerical simulation of models. However, our intention in displaying such asymptotic behavior is to show that the proposed model converges to reasonable values.

Now, we discuss results for some emblematic scenarios for the model when specific strategies are applied to Italy and France on day March 28th. For both countries, we assume $\kappa_{s}=1$ for all strategies, which means that all symptomatic individuals per day are put into quarantine. We can see that the strategy represented by the orange curves is not sufficient to significantly reduce the total number of confirmed infected individuals for France, since the last value $\theta^{eff}=1.90$ indicates a large level of social interaction in this country. On the other hand, for Italy, a considerable reduction is observed, specially for the orange-dashed curve, which indicates only the number of symptomatic cases. Strategies related to the blue curves mitigate the growth of the number of confirmed cases and, with exception of the dark blue case for Italy, lead to smaller asymptotic values when compared to the red and orange scenarios. The light blue curves, related to large social distance (θ=0.8), are the most efficient scenarios to induce an accentuated reduction of the growth and a fast convergence to the maximal number of confirmed cases. Furthermore, green curves tend to approach the medium blue curves, which means that, for θ=1.0, there is no significant difference between isolating 1% ($\kappa_{a}=0.01$) of the asymptomatic individuals per day or doing nothing. However, increasing the daily ratio of detection and isolation of asymptomatic individuals to $\kappa_{a}=0.05$, a noticeable reduction of the asymptotic value of infected individuals is observed (see magenta-dashed curves). Nevertheless, none of these strategies are better than increasing the social distance, scenario represented by the light blue curves.

**FIG. 4.**
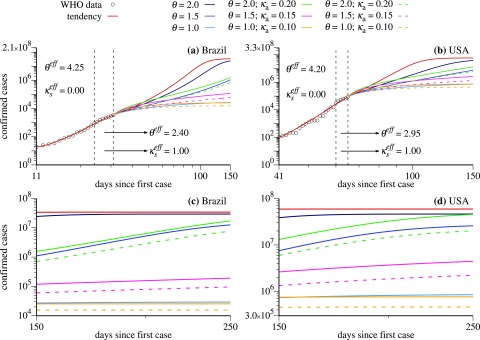
Log–log plot of cumulative number of confirmed cases (black circles) for Brazil [(a) and (c)] and USA [(b) and (d)] as a function of time and the projected number of cases (colored lines) using distinct government strategies (discussed in the text).

Next, we discuss cases for Brazil and USA using other strategies. Results are shown in Figs. 4(a)–4(d), respectively. Figures 4(c) and 4(d) furnish predictions for the number of infected individuals. Due to the distinct scenarios, we had to change the colors a bit (see also color labels in Fig. 4):

*Red curves:* have the same meaning as before.

*Blue curves:* are still related to the reduction of social interaction so that θ can take the values θ=2.0,1.5, and 1.0, going from dark blue to light blue.

*Green curves:* reduction of social interaction together with tests to identify and isolate asymptomatic and mild symptomatic cases. Here, we use θ=2.0 and $\kappa_{a}=0.20$.

*Magenta curves*: reduction of social interaction together with tests to identify and isolate asymptomatic and mild symptomatic cases. Here, we use θ=1.5 and $\kappa_{a}=0.15$.

*Orange curves*: reduction of social interaction together with tests to identify and isolate asymptomatic and mild symptomatic cases. Here, we use θ=1.0 and $\kappa_{a}=0.10$.

For the dashed curves, the parameters are the same as those from the continuous curves above but represent the total number of confirmed symptomatic individuals. We notice that asymptomatic individuals, or those with very light symptoms, would not be identified without realization of tests and are not computed in the number of confirmed cases.

As in Fig. 3, during the times for which real data are available, the model chooses the values of the parameters θ and $\kappa_{s}$ that better adjust the simulation results with the real data. In the case of Brazil and USA, we obtain two values of $\theta^{eff}$, as shown in Figs. 4(a) and 4(b) with the corresponding numerical values. Red curves fit nicely the data as long as they are available. For the USA case, there were some difficulties in adjusting the parameters since the data show some irregularities. In the case for which the strategy does not change ($\theta =\theta^{eff}$ and $\kappa_{s}=\kappa_{s}^{eff}$), the red curves increase very much for both countries. Very high asymptotic values of infected individuals are reached, $3.4\times 10^{7}$ cases for Brazil and $5.9\times 10^{7}$ cases for USA.

Now, we discuss results when new government strategies are applied to Brazil and USA on March 28th. All blue curves (dark to light) tend to mitigate the growth of the asymptotic number of cases for both countries. However, values of θ=2.0 and 1.5 still lead to large asymptotic values. The case with θ=1.0 is the most relevant strategy to flatten the curve efficiently. From the strategies that combine social distance with quarantine for the asymptomatic and mild symptomatic cases, stands out the inefficiency of the scenario with θ=2.0 and $\kappa_{a}=0.20$ to flatten the green curve. On the other hand, strategies with larger social distance (magenta and orange curves) lead to more promising scenarios. The better prediction emerges when using θ=1.0 and $\kappa_{a}=0.10$ (orange curves). For comparison, if we consider only the symptomatic cases, we obtain the asymptotic values $3.0\times 10^{4}$ (light blue curve) and $1.6\times 10^{4}$ (orange-dashed curve) for Brazil and $8.7\times 10^{5}$ (light blue curve) and $4.6\times 10^{5}$ (orange-dashed curve) for USA. As in the case of France and Italy, also for Brazil and USA, we observe that the social distance is the key element to reduce the growth rate of the maximum of symptomatic cases.

## IV. CONCLUSIONS

The power-law growth of the cumulative number of confirmed infected individuals by the COVID-19 until March 27, 2020, is shown to be the best descriptive scenario for the countries Brazil, China, France, Germany, Italy, Japan, Spain, Republic of Korea, and USA. Distinct power-law exponents for the countries are found and summarized in Table I. The power-law behavior suggests that the underlying propagation dynamics of the virus around these countries follows scale free networks, fractal kinetics, and small world features.^5,6,9^ While power-laws with distinct exponents may look visually similar, it is necessary to *quantify* this similarity. For this, we compute the Distance Correlation^10–12^ between all countries mentioned above (not shown). However, we use results for the DC from representative countries from four continents, namely, Brazil, Italy, Japan, and USA (see Fig. 2). They show that the power-law growth between these countries are highly correlated, even between North and South Hemispheres. The high correlation between the power-law curves of different continents strongly suggest that government strategies can be applied with success around the whole world.

Furthermore, we propose a variation in the well known SEIR epidemic model^19,20^ for predictions using (or not) distinct government strategies applied on March 28, 2020. We apply numerically distinct strategies to flatten (or not) the power-law curves. Even though the social isolation, a well known benefit, is very powerful to flatten the curves, we found other strategies which lead to comparable results. For Italy and France, for example, the best scenario was obtained when reducing the social interaction to θ=0.8 (light blue curves). However, if 5% of the asymptomatic and mild symptomatic individuals are identified and isolated everyday ($\kappa_{a}=0.05$), asymptotic values of the same order of magnitude for the symptomatic cases were obtained even if θ=1.0 (magenta-dashed curve). On the other hand, for Brazil and USA, our simulations confirm that to keep the social distance is essential to decrease the asymptotic number of infected individuals, and even better results could be obtained by increasing the tests to identify and isolate asymptomatic and mild symptomatic individuals (compare light blue and orange curves). The above combination between social interaction and the *huge* degree of isolation of infected individuals could be implemented to prevent economic catastrophes because people are not working. In other words, let some essential individuals go back to work (increasing θ) and, *simultaneously*, increase by a *huge* amount the number of daily tests and isolation of infected individuals. This could furnish an efficient scenario to flatten the power-law.

Nevertheless, we point out again that our main results confirm that the social isolation of individuals is by far the best efficient strategy to flatten the curves.

## Data Availability

The data that support the findings of this study are openly available from WHO (World Health Organization) (situation reports 1–68), Ref. 13.

## APPENDIX A: THE DISTANCE CORRELATION

In this section, we give a precise description of the numerical procedure to obtain the DC following Ref. 10. Consider joint random sample $\left(X,Y)=\{(X_{k},Y_{k}):k=1,\ldots ,N\right\}$ with $X,Y\in R^{p}$ and N≥2, with i=1,…,N and j=1,…,N. In addition, consider matrix $A_{ij}=a_{ij}-{\bar{a}}_{i.}-{\bar{a}}_{.j}+{\bar{a}}_{..},$ where $a_{ij}=|X_{i}-X_{j}{|}_{p}$ is the Euclidean norm of the distance between the elements of the sample; ${\bar{a}}_{i.}=\frac{1}{N}\sum_{j=1}^{N}a_{ij}$ and ${\bar{a}}_{.j}=\frac{1}{N}\sum_{i=1}^{N}a_{ij}$ are the arithmetic mean of the rows and columns, respectively; and ${\bar{a}}_{..}=\frac{1}{N^{2}}\sum_{i,j=1}^{N}a_{ij}$ is the general mean. A similar matrix, $B_{ij}=b_{ij}-{\bar{b}}_{i.}-{\bar{b}}_{.j}+{\bar{b}}_{..},$ can be defined using $b_{ij}=|Y_{i}-Y_{j}{|}_{p}$. The terms $b_{ij}$, ${\bar{b}}_{i.}$, ${\bar{b}}_{.j}$, and ${\bar{b}}_{..}$ are similar to those from matrix $A_{ij}$. From these matrices, we compute the *empirical distance correlation* from

$$DC_{N}(X,Y)=\frac{\sigma_{N}(X,Y)}{\sqrt{\sigma_{N}(X)\sigma_{N}(Y)}},$$ (A1)

where

$$\sigma_{N}(X,Y)=\frac{1}{N}\sqrt{\sum_{i,j=1}^{N}A_{ij}B_{ij}}$$ (A2)

and

$$\sigma_{N}(X)=\frac{1}{N}\sqrt{\sum_{i,j=1}^{N}A_{ij}^{2}},\;\sigma_{N}(Y)=\frac{1}{N}\sqrt{\sum_{i,j=1}^{N}B_{ij}^{2}}.$$ (A3)

## APPENDIX B: THE MODEL

The proposed model contains six ODEs and is an extension of a model known in the literature.^21–23^ Many other related models have been proposed with distinct characteristics.^20–25^ In our case, we consider symptomatic $I_{s}$ and asymptomatic $I_{a}$ infected individuals ($I_{a}$ also includes individuals with mild symptoms). Quarantine Q is also contemplated, respectively. The transition rate from asymptomatic to symptomatic cases is neglected as an first approach. The Ordinary Differential Equations (ODEs) are given by

$$\begin{array}{rl} \dot{S\;}\; & =-\frac{\theta }{T_{inf}}\frac{\left(I_{s}+\alpha I_{a}\right)}{N}S, \end{array}$$ (B1)

$$\begin{array}{rl} \dot{E\;}\; & =\frac{\theta }{T_{inf}}\frac{\left(I_{s}+\alpha I_{a}\right)}{N}S-\frac{E}{T_{lat}}, \end{array}$$ (B2)

$$\begin{array}{rl} {\dot{I\;}\;}_{s} & =(1-\beta )\frac{E}{T_{lat}}-\left(\kappa_{s}+\frac{1}{T_{inf}}\right)I_{s}, \end{array}$$ (B3)

$$\begin{array}{rl} {\dot{I\;}\;}_{a} & =\beta \frac{E}{T_{lat}}-\left(\kappa_{a}+\frac{1}{T_{inf}}\right)I_{a}, \end{array}$$ (B4)

$$\begin{array}{rl} \dot{Q\;}\; & =\kappa_{s}I_{s}+\kappa_{a}I_{a}-\frac{Q}{T_{serial}}, \end{array}$$ (B5)

$$\begin{array}{rl} \dot{R\;}\; & =\frac{I_{s}+I_{a}}{T_{inf}}+\frac{Q}{T_{serial}}. \end{array}$$ (B6)

Dot represents the time derivative and the variables are

• $N=S+E+I_{s}+I_{a}+Q+R$: total population.
• S: individuals susceptible to infection.
• E: exposed individuals, remain latent until infected.
• $I_{s}$: symptomatic individuals. Represent individuals with strong symptoms. We assume that these individuals look for health care and are included in the confirmed cases.
• $I_{a}$: asymptomatic individuals and mild symptomatic cases.
• Q: infected individuals isolated (in quarantine).
• R: individuals which were infected and not identified but became immune.

To adjust the parameters following distinct countries, as well as government measures, we used the cumulative number of confirmed infected individuals $C_{\text{\{cum\}}}$. The number of confirmed infected individuals as a function of time is defined by $C(t)=I_{s}(t)+Q(t)$. Parameters which do not depend on strategies are

• $T_{serial}=7.5$ days: mean serial interval.^26^ Is the mean time between successive cases of the transmission of the disease.
• $T_{lat}=5.2$ days: mean incubation period.^26^ Assumed to be equal to the latent time,^20^ which is the time interval the exposed individual remains infectious, also denominated incubation time.
• $T_{inf}=T_{serial}-T_{lat}$: infectious period.^20^
• α=1.0: ratio between infectiousness of asymptomatic and symptomatic individuals. We assume that the numbers of asymptomatic and symptomatic individuals are equal.
• β=0.8: population ratio which remains asymptomatic or mild symptomatic, which is the most common observed cases (see the situation report 46 in Ref. 13).

Parameters that are related to the use of distinct strategies are

• $\theta =\gamma R_{0}$: replication factor, with γ being a number that represents the proportion of interaction between individuals and $R_{0}$ the basic reproduction number. In our model, θ is an adjustable parameter according to WHO data.
• $\kappa_{s}$: isolation rate of symptomatic individuals.
• $\kappa_{a}$: isolation and identification rate of asymptomatic individuals.

In this model, no rigid quarantine is taken into account and no immunization term is defined, since until today no vaccine has been developed. In Eq. (B5), the factor $T_{serial}$ dividing Q represents a rate of exit from the quarantine (for the group R).

It is of most relevance to mention that the only adjustable parameters in our simulations are θ and $\kappa_{s}$. This is important since for systems composed of differential equations with r parameters, 2r+1 experiments are needed to obtain all the information that is potentially available about the parameters.^27^ Since in our case r=2, we need five real data to adjust parameters correctly. All real data used in Figs. 3 and 4 to find $\theta^{eff}$ and $\kappa_{s}^{eff}$ are >5. Furthermore, the initial condition for the variable $E(t_{0})$ is adjusted only in the first part of the data, where the first $\theta^{eff}$ and $\kappa_{s}^{eff}$ are determined along the data. It could be thought that such initial condition must be include in the adjustable parameters. However, even in such case, r=3 and 2r+1=7. The lowest number of available data in the first part of data is 9, as can be seen in Fig. 3(b), meaning that even in the worst case our adjustments are trustful. The goal is to minimize the mean square error between the predicted curve and real data. In the case analyzed here, $\kappa_{a}=0$ along the real data. It is only changed, not adjusted, when there are information available about the test realization in the population. We do not start the parameter’s adjustment from the first day of reported infections, but later on. The model produces better results in such cases.

Regarding the adjustment of the parameters $\left(\theta ,\kappa_{s})=(\theta^{eff},\kappa_{s}^{eff}\right)$, we minimize the mean square error separately inside the three set of data in Figs. 3(a) and 3(b) and inside the two set of data in Figs. 4(a) and 4(b). To do so, we vary the parameters inside the intervals θ∈[0,6] and $\kappa_{s}\in [0,1]$ with a step of 0.05. The initial condition for $E(t_{0})$ is determined inside the first part of the data considering $\left(\theta ,\kappa_{s},E(t_{0})\right)$ in the interval $E(t_{0})\in [0,5000]$ using a step equal to 5.

The initial conditions used in the numerical integration process of the ODEs are the following: $Q(t_{0})=R(t_{0})=0$; $I_{a}(t_{0})=\beta I_{s}(t_{0})/(1-\beta )$, where $I_{s}(t_{0})$ represent the number of confirmed infected cases, obtained from the real time-series for the day $t_{0}$. S(t) is determined accordingly to the total number of people for each country and the previous initial conditions.
